# Oleaginous Microbial Lipids’ Potential in the Prevention and Treatment of Neurological Disorders

**DOI:** 10.3390/md22020080

**Published:** 2024-02-06

**Authors:** Mariam Alhattab, Lakshana S. Moorthy, Durva Patel, Christopher M. M. Franco, Munish Puri

**Affiliations:** 1 Medical Biotechnology, College of Medicine and Public Health, Flinders University, Bedford Park, Adelaide 5042, Australia; mariam.alhattab@flinders.edu.au (M.A.); lakshana.sankaramoorthy@flinders.edu.au (L.S.M.); pate1489@flinders.edu.au (D.P.); chris.franco@flinders.edu.au (C.M.M.F.); 2 Flinders Health and Medical Research Institute, Flinders University, Adelaide 5042, Australia

**Keywords:** neuroprotection, neurological disorders, lipid nanoparticles, oleaginous microbes, omega-3 poly unsaturated fatty acids, lipid neuroprotection

## Abstract

The products of oleaginous microbes, primarily lipids, have gained tremendous attention for their health benefits in food-based applications as supplements. However, this emerging biotechnology also offers a neuroprotective treatment/management potential for various diseases that are seldom discussed. Essential fatty acids, such as DHA, are known to make up the majority of brain phospholipid membranes and are integral to cognitive function, which forms an important defense against Alzheimer’s disease. Omega-3 polyunsaturated fatty acids have also been shown to reduce recurrent epilepsy seizures and have been used in brain cancer therapies. The ratio of omega-3 to omega-6 PUFAs is essential in maintaining physiological function. Furthermore, lipids have also been employed as an effective vehicle to deliver drugs for the treatment of diseases. Lipid nanoparticle technology, used in pharmaceuticals and cosmeceuticals, has recently emerged as a biocompatible, biodegradable, low-toxicity, and high-stability means for drug delivery to address the drawbacks associated with traditional medicine delivery methods. This review aims to highlight the dual benefit that lipids offer in maintaining good health for disease prevention and in the treatment of neurological diseases.

## 1. Introduction

Neurological diseases such as Alzheimer’s disease, depression, and epilepsy affect people of all ages. It is estimated that dementia-related expenditure by the Australian government was USD 3 billion dollars in 2018–2019, with another USD 6.8 billion to provide residential aged care services to people with dementia [1]. While the global societal costs of dementia-related expenditures for treating 55.2 million people with dementia, were estimated to be USD 1313.4 billion in 2019 [2]. Where USD 210.1 billion was related to direct medical expenses, USD 656.7 billion and USD 446.5 billion were attributed to informal and long-term care, respectively [2]. To keep individuals mentally healthy and prevent or reduce the risks of some mortal disorders and neurodegenerative diseases, there has been growing research in the development of therapeutics for their treatment and management. Lipids are of interest due to the essential role of different types of lipids in neurological pathways and the health of the human brain [3].

Lipids have a unique feature as they are able to cross the blood–brain barrier (BBB). They are abundantly present in the brain and make up more than 50% of the dry weight of the brain [4]. Docosahexaenoic acid (DHA), one of the more abundant long-chain polyunsaturated fatty acids (LC-PUFA) in the brain, has been linked with the reduction of cell death and improved cognitive function [5]. Eicosapentaenoic acid (EPA) deficiencies are also thought to result in brain disorders [6], which highlights their importance in maintaining good brain function. Lipids’ ability to cross the BBB also offers a suitable vehicle for delivering drugs to target sites for combating neurological disorders [7]. As such, lipids have been explored as a means for treating/managing neurological disorders.

Today, the majority of LC-PUFAs are derived from fish sources, which are regarded as unsustainable and non-renewable. Furthermore, fish-derived sources only sustain 30% of the global demand for omega-3 supplementation [8] and are unsuitable for the growing vegan and vegetarian population [9]. The use of LC-PUFAs as a treatment for neurological diseases would further increase demand requirements, reducing the sustained portion. This necessitates an alternative sustainable and renewable plant-based source that is capable of providing the volumes required and one that caters to people’s dietary restrictions.

**Figure 1:**
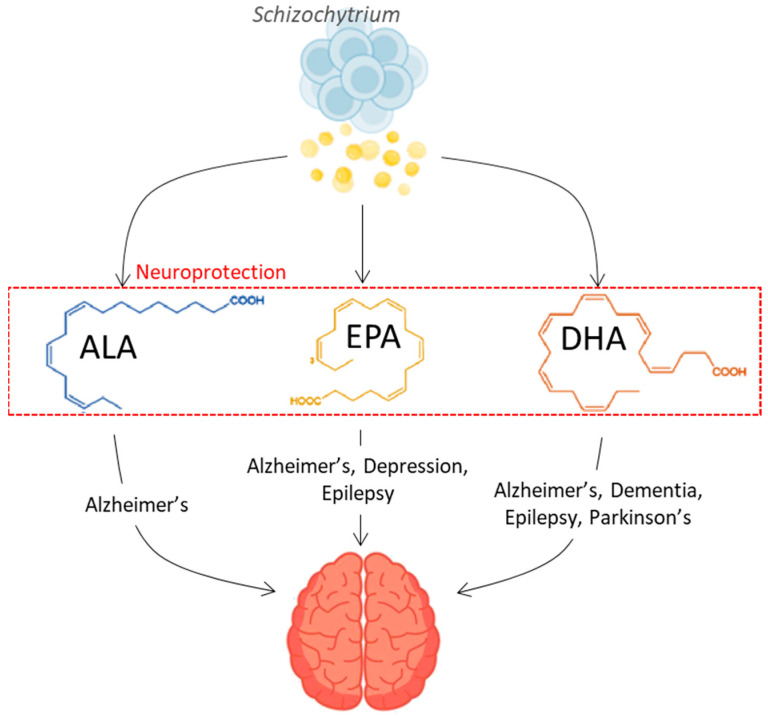
Neuroprotective potential effect of Omega-3 fatty acids derived from oleaginous organisms. ALA: α-Linolenic acid; EPA: Eicosapentaenoic acid; DHA: Docosahexaenoic acid (created with BioRender.com (accessed on 20 November 2023); Freevector [19,20]).

Oleaginous microbes, a term coined to refer to organisms with more than 20% of their weight in lipids [10], are a rich source of omega-3 and omega-6 PUFAs, as well as saturated fats. They are considered to be plant based with lipid composition capabilities of up to 20–70% of their weight [11]. They are renewable and can be tweaked to attain a desired lipid profile by altering the growth conditions, or through random mutagenesis techniques, which are considered non-genetically modifying as the modes of exposure accelerate the natural processes that would otherwise occur in the environment over time (i.e., UV exposure resembling sun radiation) [11,12,13,14]. Oleaginous microbial lipids are a rich source of long-chain fats, including saturated fatty acids (SFA) and PUFAs suitable for both dietary supplementations to combat neurological diseases and as vehicles to transport drugs across the BBB for treatment, as shown in Figure 1. Lipids play an essential role in neuroprotection and can be used as a means for preventing neurological diseases in the first instance, through dietary supplementation. Furthermore, these lipids can also be applied as vehicles for the effective transport of drugs across the BBB, delivering remedies that can treat neurological diseases. A comprehensive review of the cohort studies performed that link the relationship of omega-3 fatty acids with dementia and cognitive decline is presented elsewhere in the literature [5,15,16,17,18]. This write-up highlights the role of oleaginous microbes as an alternative renewable and sustainable source for producing plant-based lipids such as omega-3 PUFAs, as well as saturated fatty acids, and their potential use in the treatment and management of neurological diseases, which is seldomly discussed. The use of oleaginous microbial lipids would sustain current demands and cater to the growing vegan and vegetarian population. A recent review performed by Khan et al. [18] discusses the omega-3 PUFA metabolism in microalgae and their health benefits. However, here, we focus on oleaginous lipids for neurological disease prevention and treatment.

## 2. LC-PUFAs’ Role in the Treatment of Neurological Diseases

Lipids make up more than 50% of the brain’s dry weight, consisting of both structural and functional lipids such as phospholipids [4]. DHA makes up a high proportion of these lipids and 50–70% of retinal lipids [4,21]. EPA is also essential for the brain and deficiencies may result in brain-related disorders [6]. Lipids are able to cross the blood–brain barrier (BBB), and their levels in the brain have been linked to dietary intake, which makes them bioavailable for cerebral tissue [16]. There are three ways in which lipids can cross the BBB, the first of which is through the passive diffusion of fatty acids across the membranes of the endothelial cells [22]. The second and third modes of transfer are through the transcytosis pathway and the transmembrane proteins, which are discussed in detail by Pifferi, Laurent [22]. The octanol/water partition coefficient, also referred to as the Log*P* value, is used as an indicator of the BBB permeability [23]. Once the fatty acids are in the endothelium of the BBB, they are shuttled through the cytosol by binding to fatty-acid-binding proteins before being transported into the brain [22]. The prevention of neurological diseases through increased dietary intake of essential fatty acids has been explored as a means for treatment/risk reduction [16] of Alzheimer’s (AD), depression, and epilepsy [5,16,24], as they possess an important role in cognitive function and brain function, as shown in Table 1.

### 2.1. Alzheimer’s Disease

Over 55 million people were living with dementia globally in 2019 [2,38]. In the brain, DHA is abundantly present, suggesting its importance in maintaining good brain health. As we age, it is thought that the DHA levels decline, which is attributed to decreased cognitive function [5]. DHA enters the blood through ingested food and enters the brain by binding to the fatty-acid-binding protein 5 within the cerebral vascular endothelial cells [5]. Here, lysophosphatidylcholine esterifies DHA, which is preferentially absorbed into the brain rather than the free form. It accumulates in the nerve cell membrane and aids in dementia prevention [5]. DHA and its metabolites have antioxidative and anti-inflammatory properties that inhibit neuronal cell death by decreasing amyloid-beta (Aꞵ) 42 production, which is thought to improve cognitive function [5].

There is great potential for LC-PUFAs to prevent Alzheimer’s disease and aid in its treatment. Factors that have been indicated to increase the risk of developing the disease have also been identified to alter lipid metabolism. Hallmarks of AD pathology are dysfunctional neural networks and paths that may result from abnormal lipid metabolism, leading to disruption of the brain–blood barrier, abnormal processing, disturbance in cytosis, signaling, energy balance, and increased oxidation and inflammation. Furthermore, homeostatic control of lipids and transportation through apolipoprotein is important to maintain normal cognition [39].

The effect of LC-PUFA supplementation as a means to prevent Alzheimer’s disease and reduce its impact has been discussed in the literature. A recent review focused on the various intervention studies performed to assess the impact of DHA and EPA on AD concluded that the omega-3 PUFA-related improvements that were observed in experimental studies may have promoted memory formation and prevention of age-related cognitive decline [5]. It is thought that these beneficial effects may be related to a reduced risk of developing depressive symptoms as well [5,40]. In another comprehensive review of cohort studies performed, it was found that the long-term use of omega-3 fatty acid supplements in individuals resulted in a 64% reduction in Alzheimer’s disease [15]. Furthermore, DHA intake was found to reduce the risk of dementia and cognitive decline by approximately 20% [15]. However, clinical studies incorporating omega-3 PUFAs reviewed by Kerdiles et al. [16] suggested that no significant effect was observed after an Alzheimer’s disease diagnosis, but there was limited support for potential preventive effects noted. On the other hand, an observational study performed on 2612 elderly multiethnic participants, comprised of women (67%) and men aged approximately 76.3 years with a follow-up of 4.5 years, determined a lower risk of Alzheimer’s disease with increased intake of DHA and EPA [41]. The benefits of LC-PUFAs on Alzheimer’s disease and dementia were also well supported by studies exploring the effects of Mediterranean diets rich in LC-PUFAs [17,42,43,44]. Additionally, fish products and marine-derived DHA have been associated with a lower risk of AD and dementia in an earlier cohort study (including 21 studies and 181,580 participants) [45]. An observational study performed on 2612 elderly multiethnic participants, comprised of women (67%) and men aged approximately 76.3 years and with a follow-up of 4.5 years, noted a lower risk of Alzheimer’s disease with increased intake of DHA and EPA [41]. Interestingly, alpha-linolenic acid (ALA) has recently been suggested as a novel brain protector due to its role in BBB functional improvements, as the fatty acid composition of the BBB has been strongly linked with AD risk and progression [36].

These findings support the consumption of DHA-rich foods or supplementation as an essential means for continued/improved cognitive function [18,36,46]. Cell membranes with sufficient composition of LC-PUFAs have great flexibility in contrast to membranes composed of mainly saturated fatty acids and cholesterol. Certain LC-PUFAs like DHA are more abundant in the retina and brain. Their chemical reactivity and biological roles allow them to ensure cell integrity, synaptic health, and plasticity, as well as contribute to myelin synthesis and the prevention of hypoperfusion [24].

Short and medium-chain PUFAs have not shown any beneficial impacts against dementia-related disorders. The reduction in risk of Alzheimer’s disease development is mainly attributed to the role of DHA in human health, particularly in maintaining effective cognition. DHA is responsible for the optimal membrane protein interaction in signal transduction, controls gene expressions, reduces amyloid deposition, and affects cholesterol metabolism [41]. When DHA levels are reduced, dendrites are vulnerable to Aꞵ, and other lipids are used for membranes that affect their fluidity and functioning, and induce inflammatory reactions, leading to cognitive impairments [41].

### 2.2. Depression

Nutritional psychiatry, a field focusing on diet and nutrition as a remedy for therapeutic strategies that improve psychiatric disorders, is emerging [6]. Clinical studies have supported omega-3 PUFA supplementation and probiotics as a treatment option for major depressive disorder; however, further studies are needed to identify a personalized medicine approach for treating psychiatric disorders [6].

An overall beneficial effect of omega-3 PUFA supplementation on depressive symptoms was observed [18,47,48]. Higher doses of EPA especially highlighted the improvements [47,49], as well as in participants taking antidepressants alongside treatment [47]. However, in another study assessing the impact of omega-3 PUFAs as a monotherapy in adults, omega-3 supplementation was not recommended as a sole treatment, but did prove beneficial in specific populations [50]. The differences may be attributed to the different assessment criteria and doses administered in the trials. The latter study was based on 8 clinical trials assessed, out of an initial search of 96; however, the former included 13 trials from an initial search of 1955. These differences suggest that more studies are needed to provide a conclusive indicator of the effects of omega-3 PUFAs on depressive disorder. Trials need to be focused on providing more information regarding the sampling group to increase the number and administer a wide range of doses to better capture the effect spectrum. Similarly, the heterogeneity between studies was also discussed by Appleton, Voyias [51], who suggested that differences may be due to the severity of depression. With milder depressive symptoms, omega-3 PUFAs had no effect; however, more severe symptoms suggested a slight benefit [51]. The authors further assessed 35 studies and noted that omega-3 PUFA supplementation may have a small to modest benefit for depressive symptoms [51], but more complete studies were required to determine the precise effects. The study of Chang, Chang [52] also suggested that there is no beneficial effect of omega-3 supplementation on depressive symptoms; however, it did improve the core depression symptoms [52]. Despite this, the International Society for Nutritional Psychiatry Research has promoted the supplementation of omega-3 PUFAs in pregnant women, children, and elderly people with major depression disorders [6,53].

### 2.3. Epilepsy

Nearly one percent of the general population suffers from epilepsy [54]. Omega-3 PUFAs’ role in synaptic plasticity of neural membranes, immunological control in the nervous system, and protection of nerve fibres [55] has led to the thought that these fatty acids could prove beneficial in reducing recurrent seizures in epilepsy patients. In earlier research, the administration of Docosahexaenoic acid (DHA) in particular, a fatty acid that is abundantly present in brain tissues, has suggested a potential way to control seizures in both in vitro and in vivo animal models [56]. Additionally, α-linolenic acid (ALA) and linolenic acid have demonstrated beneficial effects but are not as significant as the anti-epileptic impacts of DHA [56,57,58,59], with one reason being that very low amounts of these fatty acids enter brain tissues. ALA can be saturated and elongated to form longer chains such as DHA, which could indirectly increase the seizure threshold. It can also directly alter the neuropsychiatric condition or act as a displacement for DHA from the liver and adipose tissue to suppress the seizures. It is thought that DHA may be effective due to its anti-inflammatory characteristics, which reduce the level of proinflammatory molecules like interleukin (IL)-1 β, IL-6, and tumour necrosis factor (TNF)-α that are expressed by chemically induced seizures [56]. Furthermore, triheptanoin, a triglyceride of C7 fatty acid, has displayed positive results as an anticonvulsant. Anaplerosis, which allows replenishment of the energy of the TCA (tricarboxylic acid cycle), can reduce energy failures and protect against seizure-induced cell death via the release of pyruvate through the citric cycle. When induced by mice, triheptanoin increased levels of propionyl-CoA, which is thought to produce succinyl-CoA and facilitate the refilling of the TCA cycle [60]. Hence, this suggests that several fatty acids may be of benefit to epilepsy patients. However, in recent research investigating data from nine different trials over an average period of 22 weeks, the supplementation of omega-3 PUFAs did not show a significant impact on epileptic seizures in the treated patients [61]. These varying results necessitate the need for more studies to assist in gaining a conclusive understanding.

## 3. Lipids as Delivery Vehicles for Disease Treatment

Lipids not only function to aid in neurological disease prevention, but also serve as an important tool in modern-day medicine for delivering drugs to target specific disease sites. Lipids are able to cross the blood–brain barrier, and their levels in the brain have been linked with dietary intake. Lipids consumed are bioavailable for cerebral tissue [16]. In neuropharmacology, the bioavailability of drugs is one of the main obstacles identified that needs to be overcome for new drug development [16,62].

Solid lipid nanoparticles (SLN) and nanostructured lipid carriers (NLC) are one of the emerging lipid-based drug delivery systems fabricated specifically to target and address the issues with traditional drug delivery systems, such as poor water solubility and bioavailability [63]. This form of delivery system can be modified to target various diseases whilst preserving the active drugs’ specificity and potency, using surface modifications and attachment of ligands [7].

### 3.1. Qualities of an Effective Drug Delivery System

The development of novel drug delivery systems is thought to have a tremendous impact on the treatment of diseases. Conventional delivery systems, such as oral tablets, capsules or ointments, have poor bioavailability and fluctuations in plasma and are not capable of controlling the release of the treatment [64]. Novel delivery systems focus on numerous modifications and features to improve their efficiency, including having high biocompatibility, biodegradability [65], low toxicity, high stability in blood and plasma, high efficiency in targeting the destination, and having desired uptake and release rates to specific cells [65,66]. Specifically, the ability of the drug to target specific diseased cells is an important quality, as it mitigates the side effects that often accompany treatments, as the drugs also impact healthy cells and tissue.

### 3.2. Lipids Used in Lipid Nanoparticle Formulation

A variety of lipid types have been used for lipid nanoparticle formulations, consisting of both solid and liquid lipids, as shown in Table 2. A comprehensive review of the lipids used in the formulation of lipid nanoparticles is discussed elsewhere [7]. There are a number of different factors that govern the ratio of solid and liquid lipids to be used to achieve an effective lipid matrix, such as the solubilization capacity of a drug, miscibility, cost of production, melting points, and stability [7]. Crystallinity and polymorphism are other important factors that affect lipid nanoparticles, whereby the crystal structure impacts the loading capacity of the carrier. Given these parameters, a range of lipids can be used in isolation or combination to achieve biologically stable nanocarriers of drugs, with triglycerides as a predominant lipid due to their distinct ability to remain solid under physiological conditions. They also have desirable solvent properties for drugs that are poorly soluble, making them ideal delivery vehicles in various cosmetics. Furthermore, many forms of triglycerides are digested into monoglycerides and free fatty acids (FFA) after drug delivery through the oral route [7]. Solid lipid nanoparticles made from triglycerides are also taste masking and non-toxic. Among triglycerides, other lipid forms have been proven effective in lipid nanocarriers, such as steroids, waxes, butter, and fatty acids [7]. Nonetheless, plant-based oils such as those derived from oleaginous microbial organisms suitable for sustaining large demands need to be exploited for designing novel formulations.

### 3.3. Lipid Vehicles in Treating Brain-Related Disorders

Central nervous system therapies are limited by the drug’s ability to cross the blood–brain barrier in order to reach the brain [67]. The use of lipid encapsulation as a means for allowing drug molecules to cross the BBB is one of the most efficient techniques for bypassing the BBB and improving bioavailability; however, this technique is limited to small drug molecules with molecular weights of less than 500 Da [68]. Despite crossing the BBB, molecules that are able to pass the BBB face expulsion back into the bloodstream by the resistance protein P-glycoprotein [69].

In the chemotherapy treatment of glioblastomas, temozolomide-loaded NLC resulted in tumour growth inhibition by 1.4 and 1.8 times that of temozolomide loaded in solid lipid nanoparticles (SLN), as shown in Table 3, and polymeric nanoparticles, respectively [70]. Similarly, the study from Dana, Yostawonkul [71] also showed that treatment of glioblastoma with garlic oil encapsulated in kernel palm oil compared to garlic oil alone showed a reduction in tumour cell viability of 11.9%, as compared to 90.2% with garlic oil alone [71]. In the treatment of epilepsy, carbamazepine delivery using nanostructured lipid carriers improved by 1.35–5 folds in comparison with dispersion and enhanced the accumulation in the brain through delivery across the BBB via NLC [72]. The area under the concentration–time curve in the brain was 520.4 and 244.9 µg h/mL when carbamazepine was delivered using NLC and via dispersion, respectively [72]. The oils used for NLC and SLC are mainly composed of saturated fatty acids or unsaturated fatty acids, with a minimal number of double bonds.

### 3.4. Brain Cancer Treatment Using Lipid Nanoparticle Vehicles

The various forms of chemotherapy and surgical resection remain the norm for treating brain cancers [65]. These treatments are accompanied by a number of limitations, such as non-specific distribution in serum, short time in blood circulation, and development of drug resistance, thus igniting the push to develop nanotechnology to overcome such challenges [73]. A potential solution that has gained recognition in recent years is tailoring the size, shape, and surface of nanoparticles to be effective in the treatment of various tumours.

Tumours have hallmark characteristics that make treating them difficult, such as leaky vasculature and ineffective lymphatic drainage. While these characteristics facilitate the passive accumulation of nanoparticles initially, the active accumulation is intended to be most effective in improving the binding affinity of drugs and specificity for tumour cells. To accomplish this, the surface of nanoparticles can be altered using ligands that bind the receptors that are overexpressed in cancer cells [73].

Functionalising nanoparticles with ligands is an area of rapid research development in which ligands (such as antibodies, peptides, and polysaccharides) are conjugated onto tumour-targeting nanoparticles. Functionalisation is the process of altering the surface of nanoparticles to enhance physiochemical properties and tumour-targeting accuracy. Antibodies have gained the most recognition due to their distinct specificity and favourable in vivo properties. When combining antibodies into nanoparticles, the size of the nanoparticle increases by about 40 nm. Smaller-sized nanoparticles are favoured due to their deeper penetration into tumours; thus, antibody fragments are used in place of whole antibodies to offer a smaller and more effective alternative [73].

In cancer treatment, liposomes are the preferred carriers of antibody-conjugated drug-loaded nanoparticles in cancer treatment. Cancer therapies using antibody-functionalised liposomes can be subdivided into two major classes; angiogenesis-associated targeting and uncontrolled cell- proliferation targeting [73].

Angiogenesis, which is the process of the development of new blood vessels, is one of the key characteristics of tumours, where the tumour receives high levels of oxygen and nutrients to proliferate. Here, the liposomes that contain cancer treatment drugs are conjugated to antibodies, and this helps the liposomes bind to the receptors of overexpressed receptor cells, aiding with both cytotoxicity and antiangiogenic effects that improve the effectiveness of treatment. This technique was explored by Shein, Nukolova [74], who investigated the active targeting of liposomes in brain tumours using monoclonal antibodies (mAb) against vascular endothelial growth factors (VEGF) [74]. The study concluded that antibody-conjugated liposomes facilitated higher uptake in tumour cells, leading to higher cytotoxicity when compared to non-specific and non-targeted nanoparticle treatments. On the other hand, uncontrolled cell proliferation targeting involves antibody-functionalised liposomes directed against receptors that facilitate cancer cell proliferation. This subclass of treatment is especially useful in metastatic cancers or tumours that lack blood vessels.

Tripalmitin, a lipid consisting of 16 carbon atoms, has been used as a carrier for the delivery of the chemotherapy drugs Paclitaxel and Etoposide, which are used to manage and treat various types of cancers [7,65]. Tristearin is another C18 triglyceride that was used to effectively deliver the chemotherapy treatment drugs 5-fluorouracil, doxorubicin, tamoxifen, and mitomycin [75,76]. Omega-3 PUFA supplementation has also been linked with an increased effectiveness in cancer chemotherapy drug treatment [75,77].

A recent review covering the lipid nanoparticle delivery systems that have been employed for cancer treatment, which covers brain cancer, their methods of formulation, and the administration route, are presented by Graván, Aguilera-Garrido [65]. A comprehensive review of the lipids used in the formulation of lipid nanoparticles is presented in [7]. However, the lipid sources used for the nanoparticle drug delivery formulation are seldomly discussed. This requires further consideration as lipids have been noted to prevent particular neurological diseases and have been incorporated into diets to improve brain enzymes to combat these diseases, as noted above. LC-PUFAs play a key role in the brain and are essential for good brain health. This is the first line of defense in which lipids can be used, the second of which is its solubility in the blood–brain barrier, making it a key utility in delivering drugs to combat brain diseases. The latter characteristic is one of the main reasons lipids are used as a drug delivery vehicle. However, their potential dual purpose in preventing and aiding in the treatment of diseases through effective transport across the BBB is seldomly discussed.

### 3.5. Genetic Disease Lipid Nanoparticle Treatment

Drugs that use genetic materials such as small interfering RNA (siRNA), plasmid DNA or mRNA provide potential therapies for genetic diseases, some forms of cancers, cystic fibrosis, etc., by silencing the pathological genes or by expressing therapeutic proteins [78]. These drugs can only be used within a clinical setting if they are able to be delivered effectively, due to their rapid degradation in serum, failure to accumulate in a target tissue, inability to penetrate into the target cells, and uptake by the immune system, which can easily detect and destroy vectors containing genetic information. Lipid nanoparticles are the lead non-viral delivery systems used for genetic drugs, with four LNP-based siRNA drugs in phase III trials that are on the trajectory of entering clinics [78].

## 4. Sources of LC-PUFA

At present, finite marine fish are the primary commercial source of omega-3 fatty acids, which only cater to 30% of the global demand [8]. Despite this, fish-derived sources are unsuitable for the growing vegan and vegetarian population [9]. The major omega-3 fatty acid found in plant-derived diets is ALA [79], which does not provide the essential fatty acids necessary for good brain health. These necessitate the need for not only alternative sources, but also plant-based sustainable sources [77,80].

Microalgae and several families of microorganisms including fungi and bacteria have been noted to store large amounts of lipids, with contents making up to 20–70% of their biomass, as shown in Table 4 [81,82]. Yeast lipid sources consist mainly of triglycerides and can be made up of more than 78% unsaturated fatty acids [83].

Oleaginous organisms are a promising feedstock for lipid production [84] as they are renewable, simple to culture, require little space for production, have short generation times, have high lipid productivity [83,85], and are considered plant based. Lipids are capable of preventing and aiding in the treatment of neurological diseases, as discussed above. Oleaginous organisms are an excellent source of long-chain PUFAs, such as DHA, EPA, and ALA, as shown in Table 1, Table 4 and Table 5, which have been linked with improved cognitive function, aiding in preventing Alzheimer’s disease and dementia, as they are able to cross the BBB. Species such as *Schizochytrium* sp. have been reported to produce 36% of their total lipids as DHA, amounting to 6 g/L [86], which are comparable with salmon and trout DHA levels that range from 3.3–5.8 and 3.1 g/kg (wet weight), respectively [87]. Other main microbial producers of DHA include *Aurantiochytrium* sp. with contents of 18–50% [29,30], and Thraustochytrium sp. making up 45% of its total fatty acids [26,88]. DHA levels have also been reported to make up more than 20% of the total fatty acids in other species, which include *Amphidinium* sp., *Prorocentrum triestinum* [89], *Alexandrium sanguineas*, *Heterocapsa tricuetra*, *Isochrysis galbana, Isochrysis galbana*, and *Scripsiella trochoidea* [90]. The species *Aurantiochytrium* is also a great producer of saturated fatty acids (SFA), with its contents reported to make up 75% of the total fatty acids [30]. SFAs have been employed as nanostructured carriers to effectively deliver drugs for the treatment of diseases [7]. *Mortierella* sp. is able to produce ALA, EPA, and DHA in the range of 3.9 to 5.6% of the total fatty acids, which would amount to 0.37–0.16 g/L [9], as compared to salmon EPA and DHA levels, which range from 5–12 g/kg (wet weight) [87]. Although this is a lower amount, *Mortierella* sp produces other fatty acids, which are beneficial for human health and commercially produced for infant formula [91]. However, organisms such as *Yarrowia lipolytica* and *Nannochloropsis* sp. are better sources for EPA production, with contents making up 58% and 20.5%, respectively, of the total fatty acids [33,35,92]. Other notable organisms with lipid profiles consisting of more 20% EPA include *Asterionella* sp. [89], *Alexandrium sanguinea, Heterosigma akasiwo, Chlorella ellipsoidea, Pavlova gyrans, Phaedacturum tricornutum, Skeletonema costatum* [90], *Nitschia ovalis* [93], *Porprhirudium cruentrum,* and *Tribonema* sp. [94]. While ALA producers include *Chlorella vulgaris* with levels of 8.2% of the total fatty acids [95], the fungi Mucor flavus consisting of up to 13% of its total fatty acids [37], and *Penicillium* sp. with reported fatty acid composition values of up to 7.6% [96]. Interestingly, *Desmodesmus* sp. was also reported to produce enhanced levels of ALA that reached 44% of the total fatty acids, as a result of low temperature and UV treatment [97].

**Table 4:** Lipid composition of oleaginous microorganisms.

Species	Lipid (% of Dry Weight)	References
Microalgae		[10,37,82,98,99,100,101,102,103,104]
*Schizochytrium* sp.	50–70	
*Botryococcus braunii*	25–75	
*Nannochloropsis* sp.	31–68	
*Scenedesmus* sp.	34	
*Chlorella* sp.	49–76	
Bacteria		
*Arthrobacter* sp.	>40	
*Acinetobacter calcoaceticus*	27–38	
*Rhodococcus opacus*	14–70	
Yeast		
*Cryptococcus albidus*	65	
*Rhodotorula glutinis*	72	
*Trichosporon fermentans*	36	
Fungi		
*Humicola lanuginose*	75	
*Microsphaeropsis*	22	
*M. alpina*	31	
*Mortierella isabellina*	86	
*Mucor flavus*	20	

**Table 5:** Oleaginous microbe lipid profile composition (% of total FA).

FA	*Schizochytrium* sp.	*Aurantiochytrium* sp.	*Mortierella* sp.	*Ulkenia* sp.	*Nannochloropsis oceanica*	*Chlorella vulgaris*
Myristic acid (C14:0)	15.5	4.1	0.5	1.1	5.5	0.75
Palmitic acid (C16:0)	25.2	59.8	N	27.6	21.6	27.9
Palmitioleic acid (C16:1)	0.6	N	10.7	N	24.0	N
Stearic acid (C18:0)	0.3	1.8	5.1	4.4	0.4	4.5
Linoleic acid (C18:2)	N	0.3	6.7	1.7	N	33.2
Oleic acid (C18:1)	0.7	0.87	6.2	10.5	1.8	19.7
Linolenic acid (C18:3)	N	N	5.6	8.8	0.9	8.2
Arachidonic acid (C20:4)	N	N	53.8	6.1	4.0	N
Eicosapentaenoic acid (C20:5)	N	N	4.9	5.0	20.5	N
Docosapentaenoic acid (C22:5)	8.7	4.69	N	12.4	N	N
Docosahexaenoic acid (C22:6)	36.2	19	3.9	13.7	N	0.5
Other minor FAs	12.8	10.1	2.8	8.7	0.7	4.9
SFA	41.0	75.5	N	33.3	27.5	35.6
PUFA	44.9	24.5	N	46.0	37.9	41.9
Reference	[86]	[30]	[9]	[105]	[33]	[95]

FA: fatty acid; SFA: saturated fatty acids; PUFA: polyunsaturated fatty acid; N: not noted.

Furthermore, these organisms also possess other types of lipids, Table 5, which can be used to produce other products [84] such as ARA from *Mortierella*, a fatty acid essential to infant brain development. As such, lipids derived from oleaginous microorganisms could offer a dual role in both delivering drugs across the BBB for neurological disease treatment and supplying the types of lipids needed to prevent these diseases and aid in their treatment.

### 4.1. Microalgae Growth

Microalgae are resilient and capable of growing in different conditions including autotrophic, heterotrophic, and mixotrophic culture conditions [106,107]. They do not require fertile land for their growth and can obtain their nutrients from water streams, which are otherwise considered waste [108,109,110,111]. In this regard, they have been used as a means of wastewater treatment [112,113,114]. Nutrient availability, temperature, pH, and salinity all influence the growth and lipid yields, as well as the lipid fatty acid profile [115]. In fact, nutritional stress and altering the operational parameters during cultivation are used as effective means to alter the lipid fatty acid profile [116,117] to suit industrial production.

### 4.2. Lipid Extraction

Lipid extractions can be performed using various physical and chemical techniques, where the effectiveness of the recovery is dependent on the cell wall disruption [118,119,120]. The most commonly used extraction methods are the Bligh–Dyer and Folch solvent extraction techniques [118], which use a mixture of chloroform and methanol to solubilize the lipids, separating them from the residual biomass. To further improve the lipid recoveries, various cell wall disruption pre-treatment techniques have been employed which include bead mill, ultrasound, high-pressure homogenization, microwave, and steam explosion, where the effectiveness of the technique is dependent on the cell wall composition and structure [118,119].

### 4.3. Commercial Producers

The commercial production of vegan EPA and DHA is derived from Thraustochytrids belonging to the genera *Schizochytriumm*, *Ulkenia, Aurantiochytrium*, and *Crypthecodinium*, as shown in Table 4 [121,122,123,124]. A major producer of DHA from *Schizochytriumm* and *Crypthecodinium* is DSM enterprise, which supplies algae oil consisting of 50% EPA/DHA [125]. Similarly, Solazyme Bunge Renewable Oils produces the whole of the biomass of *Schizochytriumm*, which is rich in DHA; however, it primarily caters to aquaculture feed [125]. Lonza is another producer and retailer of DHA in oil and powder-based forms that is derived from the heterotrophic organism *Ulkenia* sp. [125].

Although the DHA production from *Schizochytrium* was assessed to be greater in cost compared to fish oil by Zhang, Li [21], the authors noted that a biorefinery approach to producing various other by-products could improve the economics, which was also noted by others [123,126]. LC-PUFAs may be used both to transport drugs using lipid nanotechnology and to supply the essential lipid for disease prevention/management, which would cater to both the supplementation and pharmaceutical markets. Oleaginous microbes also possess other metabolites such as carotenoids, polyphenols, amino acids, and polysaccharides, which have been discussed as a way to prevent neurodegenerative disorders elsewhere [127]. Furthermore, fish oil sources do not cater to the growing vegan and vegetarian population, which must be considered. The global omega-3 supplementation market size in 2021 was valued at USD 6.03 billion and is expected to reach USD 10.8 billion in 2028 [128], while the lipid nanoparticle market size was valued at USD 887.2 million dollars in 2023 and is projected to reach USD 3.17 billion in the next 10 years [129]. In addition, the lipids attained from oleaginous microbes are natural sources, unlike synthetic molecules, which are less desirable.

## 5. Oleaginous Microbial Organisms as a Sustainable PUFA Source for Treatment and Management of Diseases Future Directions

Although a consensus seems to exist that LC-PUFAs exert a positive impact in preventing/aiding in the treatment of neurological diseases, such as Alzheimer’s, depression, and epilepsy, further studies are needed to provide conclusive evidence into the impact of these lipids not just on the above-mentioned disorders, but also in other neurological diseases. The vital roles that LC-PUFAs have been linked to play in aiding in the treatment of neurological disorders, as described above, are a key indicator of how essential these PUFAs are for good brain health. Furthermore, our understanding of the structural and biochemical roles of lipids in the BBB is limited [36], and further research in this area is necessary to identify how lipids can aid in combating neurological diseases. Oleaginous microbes, possessing the majority of their weight in lipids, are a promising renewable feedstock for the production of LC-PUFAs needed for neurological disease treatment and management, which are seldomly discussed [127]. The major advantage of oleaginous microbial lipids, such as that from *Schizochytrium*, is that it is considered a plant source that accommodates the growing vegan and vegetarian population and has gained the status of being generally recognized as safe (GRAS) [27]. As such, more research needs to be performed that investigates the impact of oleaginous microbial oil in the treatment and management of neurological diseases through its incorporation as a dietary supplement and use in drug delivery to combat these disorders. These studies are vital for assessing the biorefinery potential of microbes, which is needed for understanding the economic potential of this emerging biotechnology [130].

As highlighted above, lipids also offer an effective means for drug delivery across the blood–brain barrier to treat and manage neurological diseases. However, studies investigating oleaginous microbe-derived lipids as vehicles for transport are very limited [7]. Oleaginous organisms offer a wide array of lipid types consisting of saturated, monounsaturated, and polyunsaturated fats that resemble those that have been explored for the effective delivery of lipophilic drugs [7]. Thus, the lipid produced offers a dual potential as a vehicle for delivering lipophilic drugs to treat neurological diseases and LC-PUFAs that are capable of aiding in their prevention. Future studies are needed to instigate the use of these oleaginous lipids as delivery vehicles for drugs to treat neurological diseases, as they are renewable and sustainable with efficient means for lipid production compared to other marine organisms.

Microbial lipids may also be tweaked to provide a profile rich in saturated fats or long-chain PUFAs through alteration of the growth conditions or metabolic engineering approaches. Random mutagenesis and adaptive laboratory evolution are the more favourable approaches, as they are not considered genetically modifying [14]. Studies identifying the role of different fatty acids in the treatment of diseases would aid in focusing research efforts on identifying suitable metabolic engineering approaches for oleaginous microbes to enhance the production of particular fats.

Food fortification with omega-3 PUFAs has become a popular means for improving the health benefits of common everyday foods such as bread, beverages, and baked goods [18,131]. The neuroprotective benefits of these functional foods need to be investigated through experimental trials to determine their impact and the daily quantities required to gain the benefits [18]. The use of oleaginous microbial oil rich in omega-3 PUFAs would be a sustainable and renewable means for providing omega-3 lipids for food fortification as well as other beneficial metabolites.

## Figures and Tables

**Table 1:** Fatty acid treatment/management of neurological disorders and potential oleaginous microbial source.

FA (Log*P*) *	Function	Disease Treatment/Prevention	Potential Oleaginous Microbe Source of FA	Lipid Content (%)	PUFA (%)	Reference
DHA (6.78)	Brain development, cognitive function	Alzheimer’s, Dementia, Epilepsy, Parkinson’s	*Thrustochytrium* sp. T18	46.9–50	35–45 (DHA)	[5,15,21,25,26]
			*Schizochytrium* sp.	50–77	35–40 (DHA)	[10,27,28]
			*Aurantiochytrium* sp.	44–55	18–50 (DHA)	[29,30]
EPA (6.23)	Vision loss prevention, cognitive function	Alzheimer’s, Depression, Epilepsy	*Schizochytrium* sp.	50–77	1.26–7.63 (EPA)	[5,28,31]
			*Mortierella alpina*	31.5	26.7 (EPA)	[32]
			*Nannochloropsis oceania*	13.5–35	20.5 (EPA)	[33,34]
			*Yarrowia lipolytica*	30	58% (EPA)	[35]
ALA (6.50)	BBB functional improvement	Alzheimer’s	*Mortierella* sp.	28–41	5.6 (ALA)	[9,36]
			*Mucor flavus*	20	10–13 (ALA)	[37]

FA: fatty acid; DHA: Docosahexaenoic acid; EPA: Eicosapentaenoic acid; ALA: alpha-linolenic acid; BBB: blood–brain barrier; * Log*P* values, the octanol/water partition coefficient, used as an indicator of the BBB permeability [23].

**Table 2:** Solid and liquid lipid types used in lipid nanoparticle formulations (adapted from [7]).

Category	Type
Triglycerides	Trimyristin (Dynasan 114), Tripalmitin (Dynasan 116), Tristearin (Dynasan 118)
Fatty Acids	Lauric Acid (C12:0), Tetradecanoic Acid (C14:0), Palmitic Acid (C16:0), Stearic Acid (C18:0)
Fatty Alcohols	Cetyl Alcohol, Stearyl Alcohol
Glycerides	Glyceryl Behenate, Glyceryl Palitostearate, Glyceryl Stearate
Steroids	Cholesterol
Waxes	Bees Wax, Shellac Wax, Carnauba Wax
Butter/Fats	Shea Butter, Cocoa Butter, Ucuuba fat, Goat fat, Guggul lipid
Liquid Lipids (Oils)	Corn, Garlic, Argan, Sesame, Olive, Coconut, Almond, Linseed, Soybean, Watermelon, Black and Grape Seed, Castor, Rambutan, Oleic, Squalene

**Table 3:** Lipids used as vehicles for the transport of drugs in brain-related disorders.

Disease	Drug	Lipid Type	Findings	Reference
Glioblastoma	Temozolomide	SLN-Stearic acid	Tumour inhibition 1.8 times less effective compared to SLN	[70]
		NLC-glyceryl behenate	Greatest tumour inhibition	
		PNP-poly-(lactic-co-glycolic acid)	Tumour inhibition 1.8 times less effective compared to NLC	
	Garlic oil	NLC-Kernel palm oil	11.9% tumour viability	[71]
		Garlic oil	90.2% tumour cell viability	
Epilepsy	Carbamazepine	NLC-Trilaurin and oleic acid	520.4 µg h/mL	[72]
		Carbamazepine dispersion	244.9 4 µg h/mL	

SLN: solid lipid nanoparticle; NLC: nanostructured lipid carries.
